# Pressure-dependent magnetization and magnetoresistivity studies on tetragonal FeS (mackinawite): revealing its intrinsic metallic character

**DOI:** 10.1088/1468-6996/15/5/055007

**Published:** 2014-10-13

**Authors:** S J Denholme, H Okazaki, S Demura, K Deguchi, M Fujioka, T Yamaguchi, H Takeya, M ElMassalami, H Fujiwara, T Wakita, T Yokoya, Y Takano

**Affiliations:** 1 National Institute for Materials Science, 1-2-1, Sengen, Tsukuba, 305-0047, Japan; 2 Instituto de Física, Universidade Federal do Rio de Janeiro, Caixa Postal 68528, 21941-972 Rio de Janeiro RJ, Brazil; 3 Research Laboratory for Surface Science, Okayama University, Okayama, 700-8530, Japan

**Keywords:** pnictides, chalcogenides, localization effects, disordered solids

## Abstract

The transport and magnetic properties of the tetragonal Fe
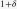
S were investigated using magnetoresistivity and magnetization within 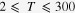 K, 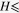 70 kOe and 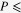 3.0 GPa. In addition, room-temperature x-ray diffraction and photoelectron spectroscopy were also applied. In contrast to previously reported nonmetallic character, Fe
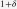
S is intrinsically metallic but due to a presence of a weak localization such metallic character is not exhibited below room temperature. An applied pressure reduces strongly this additional resistive contribution and as such enhances the temperature range of the metallic character which, for ∼3 GPa, is evident down to 75 K. The absence of superconductivity as well as the mechanism behind the weak localization will be discussed.

## 1. Introduction

Although the isomorphous Fe-based chalcogenides Fe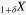 (*X* = Te, Se, S) crystallize at room-temperature to the tetragonal 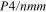 structure, however (as far as the *low-temperature* structural, magnetic, and electronic properties are concerned) their phase diagrams [1–7] are distinctly different: Fe
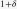
Se is an orthorhombic nonmagnetic superconductor; Fe
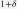
Te is a monoclinic antiferromagnetic metal while Fe
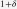
S is a tetragonal nonmagnetic and nonconducting compound [8–10] though, in sharp contrast, most theoretical work suggests metallic character [11–13]. Further distinction among these Fe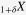 is evident in the response of their individual states to applied pressure, doping, intercalation, or a magnetic field. Most striking are the differences among the superconducting phase diagrams of their solid solutions, e.g. Fe
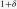
(Te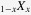) (*X* = Se, S) [1, 2]: substitution leads to a gradual suppression of magnetism and to an eventual surge of superconductivity; on the other hand, for Fe
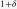
(Se
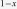
S_*x*_), substitution leads to a slight enhancement in *T*_*c*_ up to *x* = 0.2 but on further substitution the superconducting transition is monotonically suppressed.

It is remarkable that in spite of such a distinction between the electronic states of these Fe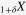 compounds, theoretical studies [11–13] predicted a metallic normal-state: while this metallicity is established for *low-temperature* phases of 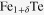 and Fe
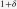
Se, experimentally Fe
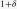
S was reported to manifest an absence of metallic conductivity [8]. Given that the question of the electronic character of Fe
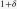
S is of a fundamental importance to the general understanding of the normal and superconducting phase diagrams of these Fe-based chalcogenides, this work addresses the electronic properties of Fe
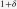
S using x-ray diffraction, spectroscopic (ultra violet photoelectron spectroscopy, UPS), and thermodynamic (magnetoresistivity and magnetization over a wide range of temperature *T*, pressure *P*, and magnetic field *H*) techniques.

Based on the stoichiometry of the iron monosulfides FeS, there are, in general, three classes [9]: (i) this Fe
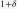
S system (mackinawite) which, just as for the other isomorphous Fe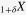, manifests an excess of Fe and crystallizes in the layered anti-PbO type structure [14]; an application of 3.3 GPa at room temperature transforms its tetragonal phase into an orthorhombic structure [15]. (ii) the near-stoichiometric and hexagonal antiferromagnetic FeS (troilite) [16, 17], and (iii) the hexagonal Fe–deficient ferromagnetic Fe
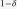
S (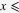 0.2) which crystallizes in the nickel arsenide form (pyrrohotite) [18]. The structural and physical properties of both FeS and Fe
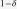
S have been extensively investigated [18, 19]; in contrast, Fe
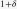
S has been relatively unexplored except for some structural and mineralogical studies [20–22]: neutron diffraction and Mössbauer analysis indicated nonmagnetic character [8]; this contradicts an analysis done by photoelectron spectroscopy (PES) [13] which suggested, instead, a single-stripe antiferromagnetic ground state.

Electronic structure calculations [11–13, 23] on Fe
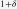
S indicated a significant Fe 3*d* orbital delocalization (primarily due to the basal-plane, intralayer Fe-Fe interactions), a dominant 3*d* contribution to the density of states (DOS, 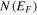) at the Fermi level, *E*_*F*_, and a weaker hybridization between the Fe and S [11–13]. As mentioned above such a predicted metallic character is in disagreement with the experimentally observed nonmetallicity. In this work, we show that Fe
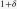
S is indeed metallic just as theoretically predicted; the reported nonmetallicity [8, 24] will be shown to be due to a localization of charge carriers. It is recalled that such a discrepancy between experiment and theory had already been reported in other transition metal sulfides [25]: e.g., troilite is a p-type semiconductor with a band gap of 0.04 eV [17]; yet band structure calculations have placed *E*_*F*_ within the *d*-*p* hybridized bands [26]. Similarly, a PES study on pyrrohotite reported a 25–30% narrower Fe 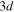 DOS band-width than the theoretical prediction [27].

## 2. Experimental

Mackinawite was synthesized using the method reported by Lennie *et al* [21]. Powder x-ray diffractograms on a conventional Cu *Kα* diffractometer indicated a single phase 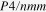 structure with *a* = 3.675(2) Å , *c* = 5.035(6) Å . Based on an energy dispersive x-ray analysis, the actual stoichiometry was found to be Fe:S = 0.52:0.48 giving Fe
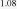
S which is in agreement with the reported ranges [21, 28].

It is well-known that the tetragonal Fe
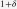
S is chemically unstable against a variation in *P*, *T* and aging [29]: aging at room temperature would slowly transform it into an amorphous product plus the semi-metallic cubic Fe_3_S_4_ (greigite). During this study, it became evident that (i) such a conversion can be temporarily inhibited if the sample is stored at cooler temperatures e.g. below 5° C; (ii) this tetragonal Fe
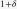
S, when subjected to a higher pressure, would start to convert into an amorphous product, reminiscent of the amorphization in Fe
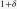
Se and FeSe
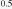
Te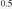 [30, 31], plus the semiconducting hexagonal Fe
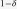
S (troilite). Accordingly, such a phase instability requires that extra care should be exercised during (as well as before and after) the measurements so as to ensure that all results had been obtained on the very same tetragonal phase: otherwise most of the results (in particular the resistivity) are irreproducible. With this in mind, the following measurements and their analysis were carried out.

Resistivity, *ρ*, was measured using a standard four-in-line method on cold-pressed pellets (care was undertaken to ensure that the grain boundary influence was minimized—see below). For 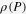, hydrostatic pressures, up to 3.0 GPa, were generated by a BeCu/NiCrAl clamped piston-cylinder cell using Fluorinert as a *P*-transmitting fluid while Pb as a manometer. Similarly, *P*-dependent magnetizations were measured using a hydrostatic pressure cell (up to 1 GPa). Daphne oil was used as a *P*-transmitting fluid while Sn as a manometer.

UPS was measured at a base pressure of 2.0 × 10^−8^ Pa and at a temperature of 300 K with He I (21.2 eV), He II (40.8 eV) and Xe I (8.44 eV) resonance lines. So as to obtain a fresh surface, samples were cut within an ultra-high vacuum chamber. The Fermi energy was referenced to that of an Au film which was measured frequently during the experiments.

## 3. Results

**Figure 1.**
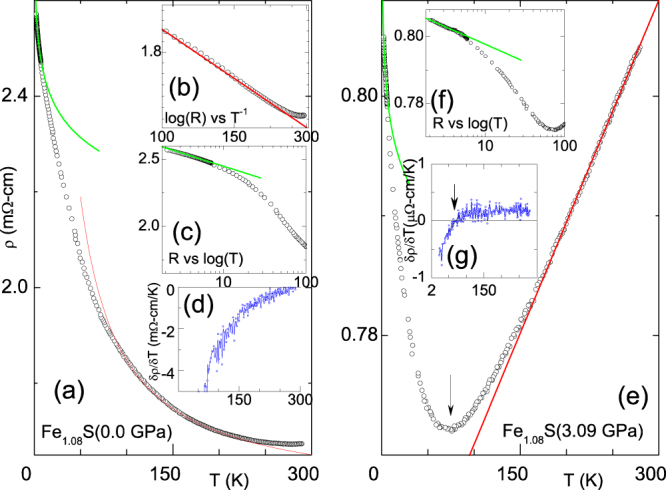
Typical resistivity curves of Fe
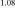
s (a)–(d) under ambient-pressure while (e)–(g) are under an applied pressure of 3.09 GPa. (a) and (e) isobaric *ρ* 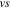 *T* curves. (b) *ρ* 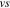 *T* curve in a log-reciprocal plot emphasizing the activated process; the solid line is a fit to equation (1). (c) and (f) *ρ* 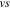 *T* curves in a linear-log plot emphasizing the weak localization behavior; the solid lines is a fit to equation (2). (d) and (g) 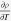 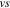 *T* curve: a negative (positive) value represents nonmetallic- (metallic-) like behavior. The crossover point is denoted as *T*_*L*_. The obtained 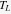, 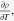 *Δ*, *S* parameters are collected in figure 2.

Figure1(a) illustrates 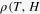 = 0, *P* = 0.1 MPa*)* of Fe
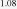
S [8, 24]. It is remarkable that 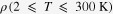 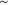 m*Ω*-cm suggesting that this (monotonic but non-sharp) low-*T* rise is not due to a conventional metal-insulator transition; most probably, it is a manifestation of localization of charge carriers [32–36] (see below). Following the analysis of [37], it is taken that the resistivity is intrinsically metallic, any nonmetallicity is attributed to this localization. Figure 1(d) reveals such nonmetallic character as a negative 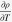. Moreover, as 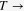 300 K, 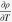 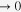 at ∼300 K: assuming a stable tetragonal phase (see Experimental), the event 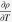 = 0 is taken as a crossover from a nonmetallic state into a metallic one. A closer look at the evolution of 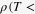 300 K*)* suggests that there are at least two types of localization-induced behavior [37]. The first appears to be a thermally-assisted behavior [38, 39]:

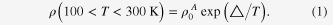
Such a thermally activated (△ ∼ 20 K) process (see figure 1(b)) is assumed to be due to a hopping of carriers from one localized state into an itinerant state which is separated by an effective energy [*E*-*E_c_*] where *E_c_* represents the mobility edge [38, 39] and should not be confused with the conventional semiconducting behavior. A manifestation of an activated behavior below a crossover/transition was already observed in RNiO_3_ [40, 41]. The second process looks like a weak-localization process which is often encountered in the low-*T* phase of a disordered metal [32–36]. In chalcogenides [37], the disorder is attributed to the nonperiodic scattering potentials (see below). Then their 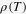 should follow [32–36]

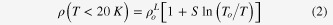
where *S* is a measure of the scattering process while *T*_*o*_ and *ρ*_*o*_^*L*^ are any measured pair (figure 1(c)). Such a log-in-*T* character was already reported for other chalcogenides [5, 37, 42, 43]. Presently it is not evident why this process is not proceeded by a metallic-like state as observed in, e.g., [37].

An application of pressure leads to pronounced effects: e.g. (i) on comparing 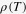 of panels (a) and (e) of figure 1, one notices a reduction in the overall resistivity, and (ii) the crossover point signalled by 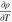 = 0, denoted as *T*_*L*_(*P*), moves to well below 300 K: the metallicity is pressure-enhanced to a wide range of temperature [40, 41].

**Figure 2.**
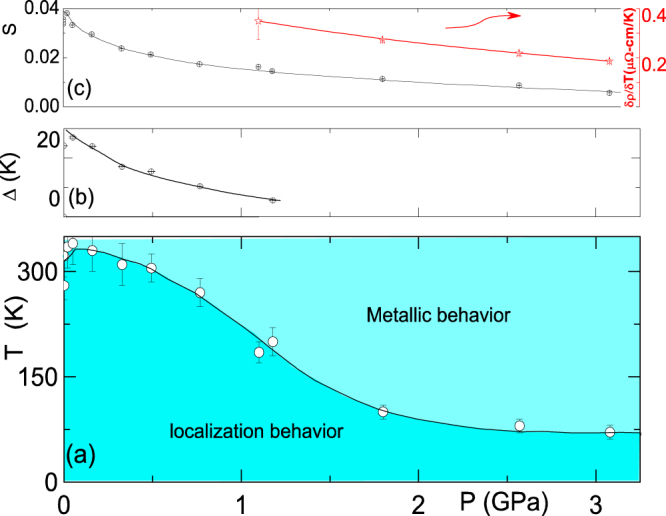
The baric evolution of (a) *T*_*L*_ of Fe
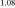
S (a measure of localization strength which is emphasized below *T*_*L*_(*P*) curve), (b) *Δ* (the activation energy in equation (1)) (c) *left ordinate:S* (as a measure of strength of the localization process below 20 K, see equation (2)); *right ordinate:* 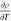 within 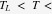 300 K (a measure of the thermal evolution of the metallic resistivity).

Just as for the ambient-pressure case, 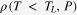 of figure 1(e) was analyzed in terms of the above mentioned two processes. The baric evolution of the fit parameters are shown in figure 2; *P* reduces all scattering processes: a monotonic decrease of (i) 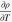 within the metallic state, (ii) *Δ* within the activated region, and (iii) *S* below 20 K. All these influences lead to a strong reduction of 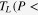 2 GPa*)*. Above 2 GPa, *T*_*L*_(*P*) is weakly but monotonically decreasing till 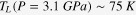; such a thermal evolution is also manifested for each of the parameters shown in figure 2(b)-(c).

figure 2(a) identifies unambiguously the metallic state as being an intrinsic high-*T* property of Fe
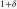
S: this provides a direct confirmation of the theoretical predictions. Evidently, without the clarification provided by the high-*P* or high-*T* 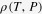 curves, the activated rise in *ρ*(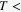 300 K) as *T* is lowered would be mistakenly taken as indicative of intrinsic nonmetallic conductivity [8–10, 24].

**Figure 3.**
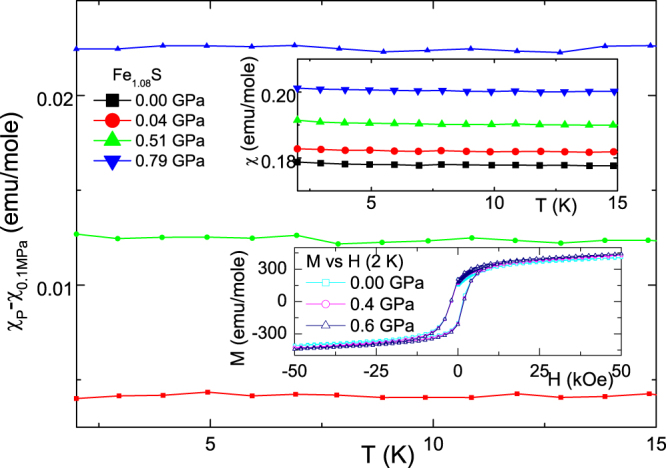
The excess, pressure-dependent molar susceptibility of Fe
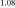
S. The ambient pressure 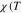,P = 0.0 GPa*)* curve (upper inset) is similar to that of Sines *et al* [44]. As the total contribution includes those of weak magnetic impurities and the cell body and as that these contributions are not influenced by *P* (see the two insets), then, for clarity, these contributions are subtracted out by plotting, in the main frame, 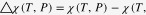 0.0 GPa*)*.

Various possible mechanisms can give rise to the pressure influence on each of the 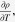 *Δ*, *S* parameters (figure 2); two of which are (i) the cold-pressed pelletizing process brings together the already metallic grains; as such an application of further pressure (during the 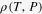 measurement) would lead to a further enhancement of the grain connectivity. (ii) The influence of the pressure is intrinsic both on the involved scattering processes as well as on the electronic structure of Fe
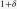
S. To differentiate between which of these is the most plausible mechanism, we carried out a magnetization measurements. Based on the above, Fe
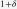
S is expected to be a nonmagnetic metal, thus its 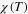 should be constant-in-*T*, Pauli-like and proportional to 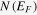. If the observed *P*-induced effects are due to grains connectivity then 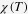 should not be influenced. If, otherwise, *P* influences its nonpolarized electronic structure, then its 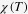 should also be modified. Indeed figure 3 indicates a *P*-induced enhancement of *χ* and as such an enhancement of 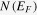: this is in an excellent agreement with the *P*-induced enhancement of the conductivity observed in figure 1.

The general features of figures 1–3 can also be interpreted in terms of a *P*-induced reduction of the involved scattering processes (not only as an enhancement of 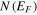 as in figure 3); this suggests that (i) within the metallic state (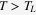), the electron-phonon or elelctron-electron interactions are reduced and that (ii) for 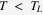, *P* induces a partial (but weak) delocalization of those carriers that had been previously localized.

**Figure 4.**
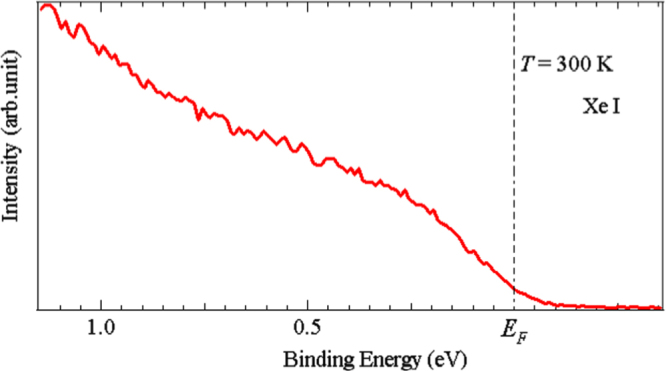
UPS spectrum of Fe
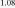
S near *E*_*F*_ (dashed vertical line) using a Xe I source (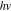 = 8.44 eV), under ultrahigh vacuum at *T* = 300 K.

From above it is concluded that Fe
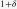
S is intrinsically metallic but below *T*_*L*_ localization effects are manifested. Then it is interesting to investigate the influence of localization on the electronic states at the Fermi surface. We addressed this question by carrying out a PES study using UPS. Fig 4 shows a UPS spectra near *E*_*F*_ measured using a Xe I source of 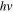 = 8.44 eV under ultrahigh vacuum at 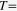 300 K. We observed a broad spectra with no Fermi edge: a non-metallic state which should be contrasted with the metallic features observed in Fe 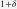 Te [45]. The presence of localization in this system could account for these features. We observed the same phenomenon with the He I (21.2 eV) and He II (40.8 eV) spectra but given the greater mean free path of the Xe I source (ca. 1 *μ*m) we take this result as being more representative of the bulk sample.

**Figure 5.**
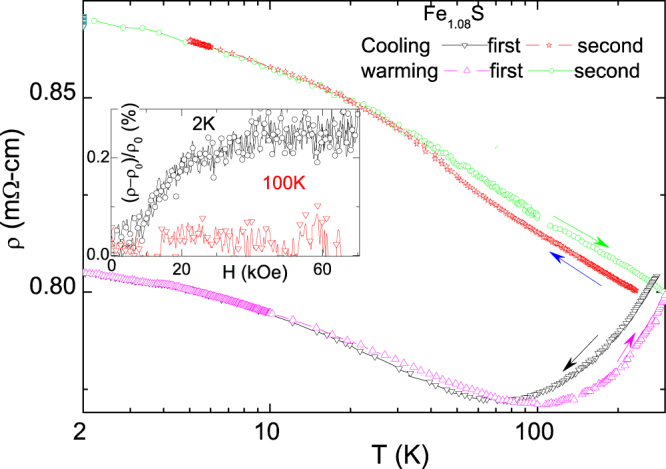
Various resistivity curves of Fe
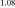
S measured under 3.0 GPa: the resistivity curve during the first cooling is similar to the first warming (taking into consideration the inherent thermal lag due to the massive body of the pressure cell). On a second cooling, the resistivity deviates strongly from the first cooling curve: this is related to phase instability. On a final warming branch, the resistivity retraces the behavior of the second cooling. The inset shows that the magnetoresistivity measured at 2 K (circles) and 100 K (triangle).

The phase instability of Fe
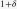
S can be best illustrated by 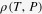 of figure 5: on a first cooling branch, 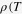,3 GPa*)* shows metallic behavior followed by a localization-induced uprise below *T*_*L*_. On warming, 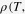 3 GPa*)* follows the cooling curve except at high-*T* wherein thermal lag is manifested due to thermal gradients that are generated across the massive body of the pressure cell. On a second cooling branch, after some days at room temperature, 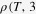 GPa) was found to be completely modified, showing an absence of metallic-like behavior and an uprise on lowering the temperature which starts already at 300 K. This irreproducibility is related to the above mentioned phase instability: indeed post-measurement XRD data indicated a partial phase transformation to the hexagonal troilite form but with no evident change in the lattice parameters of the remaining mackinawite phase. Evidence of amorphous material (most probably amorphous mackinawite) was also found.

## 4. Discussion and conclusions

Figure 2(a) is a manifestation of a pressure-induced enhancement of the stability of the metallic phase [46]. Similar enhancement had been observed in the *R*NiO_3_ charge-transfer perovskites [40, 41], wherein a metal-insulator transition at 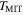 marks the sharp uprise in 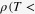 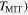 and, furthermore, the monotonic decrease in 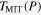 is related to the *P*-induced decrease in the charge-transfer gap. In spite of the similarity in the manifestation of the localization and the associated *P*-induced effects, we believe that the crossover event in Fe
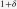
S is not due to an Anderson-type metal-insulator transition because (i) the is no low-*T* AF order or a strong hysteresis effects, (ii) the rise in 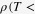 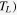 is weak (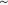 m*Ω*-cm), smooth and extends over a wider temperature range, and (iii) there are two (an activated and a log-in-*T* ) processes operating at different temperature regions. Instead, it is assumed that there is a weak localization process which is due to scattering from any non-periodically arranged potentials (the most probable disorder/defects centres are the randomly distributed excess Fe or chalcogens deficiencies as in 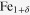 Te and Fe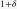 Se) [5, 37, 42, 43].

The manifestation of two types of localization processes is not unique to Fe
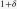
S; it had been already observed in thin films [33] though the order of appearance, as 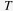 is varied, is inverted. The activated behavior (equation (1)) within 100 K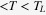 is taken to be due to a hopping of carriers; on the other hand, the log-in-*T* (equation (2)) behavior below 20 K is attributed to quantum corrections arising from scattering from the above mentioned non-periodic potentials [32, 33].

The log-in-*T* relation [32, 33] is also valid for localization of weakly interacting carriers though with a different logarithmic prefactor [34, 35]. Alternatively, interaction effects in disordered 2D Fermi systems within the metallic regime can also give rise to a log-in-*T* relation [36]. As is the usual practice, a distinction between whether a log-in-*T* behavior is due to either a non-interacting [32, 34, 35] or an interacting carriers can be obtained from a magnetoresistivity experiment: on increasing *H*, a negative magnetoresistivity is manifested for the weak localization case while a positive one for the interaction case. The magnetoresistivity of Fe
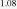
S at 2 K (inset of figure 5) is positive indicating that interactions among the diffusing carriers are important [36]. Such a manifestation of electron-electron interactions is taken to be behind the absence of superconductivity in Fe
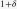
S: indeed no such strong field-dependent magnetoresistivity had been observed in the isomorphous Fe
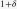
Se (see figure 5 of [47]). At higher temperature (>100 K), such a positive magnetoresistivity is drastically reduced while, at higher field, there is a tendency towards negative magnetoresistivity.

In summary, Fe
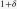
S is shown to be a metal but due to localization processes, such metallicity is not reflected in the thermal evolution of 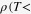 300 K*)* nor in the UPS spectra. Applied pressure does reduce the influence of the localization processes and as such the metallic character is manifested even for temperatures as low as 75 K at 3.0 GPa. Such a pressure influence is also evident in the Pauli-like susceptibility which is enhanced monotonically with *P*. Using low-*T* magnetoresistivity analysis, the weak localization that gives rise to a log-in-*T* behavior is suggested to be due to interaction effects in this disordered Fe-based system. It is assumed that such electron-electron interactions are behind the absence of superconductivity in this Fe-based chalcogenide.
